# Carbolic Acid in Surgery

**Published:** 1867-12

**Authors:** George Derby

**Affiliations:** Surgeon to the Boston City Hospital.


### ARTICLE VIII.

### *Carbolic Acid in Surgery.*

By George Derby, M.D., Surgeon to the Boston City Hospital.

The use of carbolic acid in surgery is based directly upon the investigations of M. Pasteur on putrefaction, in which he shows the relation of infusoria to the process. The results of his experiments, as published in the *Comptes Rendus* of the French Academy, and in the *Annales des Sciences Naturelles,* are briefly these :—Putrefaction* is fermentation. Fermentation is putrefaction. The process is one and the same, and in all cases determined by the presence of infusoria. They, in fact, are the true ferment. These infusoria are minutely described by Ehrenberg and also by Pasteur. They are chiefly Vibrios and Bacteria. Pasteur† says they resemble, in certain respects, plants rather than animals, and they may be regarded as among the most minute and imperfect organized forms of life, whether of the animal or vegetable kingdom.

"Should the microscopic beings disappear from the globe, the surface of the earth would be encumbered with dead organic material, and bodies of all sorts, animal and vegetable. Without them life would become impossible, since the work of death would be incomplete."‡

---

* Comptes Rendus, t. 56, p. 1189 † Ibid, t. 52, p, 1260. ‡ Ibid, t. 54, p. 265

The above extract from Pasteur shows how far these ideas of putrefaction reach. They revolutionize the doctrine that the vital principle alone defends organic material from the destructive action of oxygen, and shows that neither air nor water can convert organized substances into simple elements. That process is due to infusoria.

These organic germs are everywhere present in the atmosphere.* Pasteur found them on the Jura, at Montanvert, and at other great elevations, although in diminished numbers, In Paris, every bubble of the air contains myriads. In hospitals they particularly abound. It is certain that they exist in every place occupied by man.

The application of these ideas to practical surgery was first made by Mr. Lyster, a surgeon of Glasgow, and an account of his experiments has been recently published in the London *Lancet.* He sought to prevent decomposition of the fluids thrown out in cases of compound fracture. To do this an agent was required sufficiently powerfully to kill the infusoria already admitted by the external wound, and which should not be also destructive to the tissues. Such an agent is found in carbolic acid. This he applies freely to all lacerated parts which have been exposed to the air, and then seals up the external wound with the same substance, claiming that by this process a compound fracture is converted into a simple one. Abscesses are also treated on the same principle, the access of infusoria to their interior being prevented by holding a cloth wet with carbolic acid in front of the abscess at the moment of making an incision.

It has occured to the writer that a simpler and more effectual way of securing this result would be by throwing a jet of carbolic acid spray from an atomizer upon the point of opening.—*Boston Med. and Surg. Journal.*

---

* Annales des Sciences Naturelles, t. 16, p. 45.

